# Machine learning versus physicians’ prediction of acute kidney injury in critically ill adults: a prospective evaluation of the AKIpredictor

**DOI:** 10.1186/s13054-019-2563-x

**Published:** 2019-08-16

**Authors:** Marine Flechet, Stefano Falini, Claudia Bonetti, Fabian Güiza, Miet Schetz, Greet Van den Berghe, Geert Meyfroidt

**Affiliations:** 1 Department of Anesthesia and General Intensive Care, Humanitas Clinical and Research Center, via Manzoni 56, Rozzano, 20089 Milan, Italy; 2 University of Milano-Bicocca, Piazza dell’Ateneo Nuovo 1, 20126 Milan, Italy; 3 Clinical Division and Laboratory of Intensive Care Medicine, Academic Department of Cellular and Molecular Medicine, KU Leuven, Herestraat 49, B-3000 Leuven, Belgium

**Keywords:** Acute kidney injury, AKIpredictor, Predictive modeling, Machine learning

## Abstract

**Background:**

Early diagnosis of acute kidney injury (AKI) is a major challenge in the intensive care unit (ICU). The AKIpredictor is a set of machine-learning-based prediction models for AKI using routinely collected patient information, and accessible online. In order to evaluate its clinical value, the AKIpredictor was compared to physicians’ predictions.

**Methods:**

Prospective observational study in five ICUs of a tertiary academic center. Critically ill adults without end-stage renal disease or AKI upon admission were considered for enrollment. Using structured questionnaires, physicians were asked upon admission, on the first morning, and after 24 h to predict the development of AKI stages 2 or 3 (AKI-23) during the first week of ICU stay. Discrimination, calibration, and net benefit of physicians’ predictions were compared against the ones by the AKIpredictor.

**Results:**

Two hundred fifty-two patients were included, 30 (12%) developed AKI-23. In the cohort of patients with predictions by physicians and AKIpredictor, the performance of physicians and AKIpredictor were respectively upon ICU admission, area under the receiver operating characteristic curve (AUROC) 0.80 [0.69–0.92] versus 0.75 [0.62–0.88] (*n* = 120, *P* = 0.25) with net benefit in ranges 0–26% versus 0–74%; on the first morning, AUROC 0.94 [0.89–0.98] versus 0.89 [0.82–0.97] (*n* = 187, *P* = 0.27) with main net benefit in ranges 0–10% versus 0–48%; after 24 h, AUROC 0.95 [0.89–1.00] versus 0.89 [0.79–0.99] (*n* = 89, *P* = 0.09) with main net benefit in ranges 0–67% versus 0–50%.

**Conclusions:**

The machine-learning-based AKIpredictor achieved similar discriminative performance as physicians for prediction of AKI-23, and higher net benefit overall, because physicians overestimated the risk of AKI. This suggests an added value of the systematic risk stratification by the AKIpredictor to physicians’ predictions, in particular to select high-risk patients or reduce false positives in studies evaluating new and potentially harmful therapies. Due to the low event rate, future studies are needed to validate these findings.

**Trial registration:**

ClinicalTrials.gov, NCT03574896 registration date: July 2nd, 2018

**Electronic supplementary material:**

The online version of this article (10.1186/s13054-019-2563-x) contains supplementary material, which is available to authorized users.

## Background

Acute kidney injury (AKI) is an abrupt decline in kidney function that is highly prevalent in critically ill patients [1–3]. AKI has an unfavorable impact on both short- and long-term outcomes and is associated with increased financial costs [4–6]. The international Kidney Disease: Improving Global Outcomes (KDIGO) work group classified AKI in three stages of ascending severity [7], according to a quantitative increase in serum creatinine (SCr) or a decrease in urine output (UO). However, both are late and unspecific markers of the underlying pathological insult. The late recognition of AKI is one of the potential factors to explain the lack of evidence-based therapeutic options to prevent AKI or attenuate its course [8–10]. Early biomarkers for AKI have been proposed and showed good predictive performance in particular settings [11, 12]. However, as their measurement requires additional (expensive) lab tests, it is necessary to identify which patient subgroups would benefit most from biomarker testing.

Prediction models for AKI have also been investigated. These models have the advantage that they use the information already present in the (electronic) health records [13, 14]. The prediction score developed by Forni et al. [15] is a simple scoring system to detect hospital-acquired AKI. Recently externally validated [16], the score showed moderate discrimination and acceptable calibration. The prediction model developed by Haines and colleagues, for trauma patients admitted to critical care, demonstrated good discrimination, both for any stage of AKI (AKI-123) and for its most severe stages (AKI-23) [17]. Finally, in a general ICU population from the multi-center randomized controlled EPaNIC trial [18], the AKIpredictor models [19] were developed, with advanced machine learning techniques, to predict AKI at different time points in the clinical course of the patient (before admission, upon admission, on the first morning after admission, and after 24 h), for AKI-123 or AKI-23. The AKIpredictor, made available online at http://www.akipredictor.com, not only proved a high degree of accuracy in a separate validation cohort [19], but also outperformed serum neutrophil gelatinase-associated lipocalin, a biomarker for AKI [20, 21].

The potential usefulness of the AKIpredictor [16, 22–24] or similar prediction models [13, 25] has been recognized. However, it remains to be investigated whether and how these models can be used in clinical practice. Such models should be evaluated prospectively in new and previously unseen patient cohorts. The use of available clinical data to estimate the risk of critically ill patients to develop complications such as AKI is part of the daily practice of ICU physicians. Therefore, it can be expected that they will perform well at this task. Therefore, comparing the performance of prediction models against physicians’ predictions could add a dimension to the evaluation. In the present study, the performance of the prediction model AKIpredictor to predict AKI-23 within the first week of ICU stay was evaluated prospectively and compared against predictions by ICU physicians.

## Methods

This prospective observational study was performed during the predefined period of May and June 2018 in five ICUs of the University Hospitals Leuven, Belgium. The Institutional Review Board approved the enrollment and clinical data collection protocol, providing waiver of consent for study participation. The study is registered at ClinicalTrials.gov (NCT03574896).

### Study population

All critically ill adults consecutively admitted within the study period were eligible for the study. Patients were excluded if they had pre-existing end-stage renal disease (ESRD) or had already developed AKI upon ICU admission. In case of ICU readmission, only the first admission was considered. Additionally, patients were excluded if they had been already admitted during the previous 3 months. Patients for whom all predictive moments occurred during on-call time were excluded because of the unavailability of research staff to hand out the questionnaires. Patients with scheduled admission after surgery were brought to the ICU directly after the end of the procedure.

### Endpoint

The primary objective of the study was the comparison of the diagnostic performances of AKIpredictor, a classification prediction model for AKI, and physicians in predicting AKI-23 in the 7 days following ICU admission. Predictions were formulated upon admission (admission cohort), on the first morning of ICU stay (day1 cohort), and after 24 h of ICU stay (day1+ cohort). If AKI became evident before one of such time points, no further prediction was made. Secondary objectives were (a) to assess the influence of the level of seniority or prediction confidence on the accuracy of physicians’ predictions and (b) to compare the AKIpredictor performance using two definitions of AKI (SCr versus SCr and UO).

### Acute kidney injury

AKI was staged each day of the first week using the SCr and UO criteria from the most recent guidelines (KDIGO) [7]. For external validation of the AKIpredictor and comparison with the development study [19], where AKI was classified only by SCr, AKI was also staged each day based on the SCr criterion. Baseline SCr values were defined as the lowest SCr value identified in the 3 months prior to and not including admission. If no baseline SCr was available, it was calculated with the Modification of Diet in Renal Disease formula using an estimated glomerular filtration rate of 75 mL/min/1.73 m^2^ [26].

### AKIpredictor predictions

All predictions from the AKIpredictor are based on routinely collected patient information (Table 1). Thanks to the prospective design of the study, there was no missing value in the variables required by the AKIpredictor. Predictions were retrospectively calculated as risk percentage upon admission, on the first morning of ICU stay, and after 24 h.

**Table 1:** Variables included in the different models of the AKIpredictor [19]

Admission	Blood glucose upon ICU admission
	Suspected sepsis upon ICU admission (yes/no)
	Hemodynamic support upon ICU admission (none/mechanical/pharmacological/both)
Day1	Serum creatinine
	APACHE II score
	Maximum lactate
	Bilirubin
	Hours of ICU stay
Day1+	Total amount of urine
	Urine slope^1^
	Time the mean arterial blood pressure is above its average value
	Time the mean arterial blood pressure is below 60 mmHg
	Pharmacologic hemodynamic support (cumulative dose of inotropes and vasopressors)

The day1 model also uses the predictors of the admission model; the day1+ model also uses the predictors of the day1 and admission models

*Abbreviations*: *APACHE* Acute Physiology and Chronic Health Evaluation, *ICU* intensive care unit

^1^The urine slope refers to the slope of a linear model fitted to the hourly urine values

### Physicians’ predictions

Questionnaires (Additional file 1: Appendix A) were handed to the ICU physicians at the same target time point than the AKIpredictor: upon admission, on the first morning of ICU stay, and after 24 h. Clinicians were blinded to the AKIpredictor predictions at all time points. Prospective data collection included:

- Physicians’ binary predictions: *Do you think this patient will develop AKI stage 2 or 3 over the next 7 days? (yes-no).* Binary predictions were used to determine physicians’ classification thresholds and their derived sensitivity and specificity.
- Physicians’ prediction as percentage: *What is your prediction that this patient will develop AKI stage 2 or 3 over the next 7 days? (scale 0–100%)*
- Physicians’ level of confidence about their prediction: *How confident do you feel about this prediction? (low-medium-high)*

To accommodate for physicians’ availability, questionnaires were considered valid if collected within 1 h before up to 3 h after the predefined target time point. When several predictions were available per patient, an average of the percentage predictions was calculated.

Comparison between the AKIpredictor and physicians was only assessed in the subset of patients who had both AKI predictions measured.

Two categories of physicians were interviewed: juniors (junior residents) and seniors (senior residents and staff members) (described in Additional file 1: Supplementary methods). Their age, gender, and seniority level were recorded (Additional file 1: Appendix B). Pre-planned sub-analyses were conducted to investigate the performance by level of seniority and by level of confidence in prediction.

### Statistical analysis

Data are presented as means and standard deviations (SD), medians and interquartile ranges (IQR), and numbers and proportions where appropriate. Statistical significance was set at *P* < 0.05. All analyses were performed using Python version 2.7.13 (Python Software Foundation, http://www.python.org), Scipy version 0.18.1 (SciPy.org), and R version 3.5.0.

Reporting of the study was performed using the STROBE guidelines for observational studies [27], STARD guidelines for diagnostic test [28], and guidelines for reporting machine learning predictive modeling [29].

#### Diagnostic accuracy assessment

**Fig. 1:**
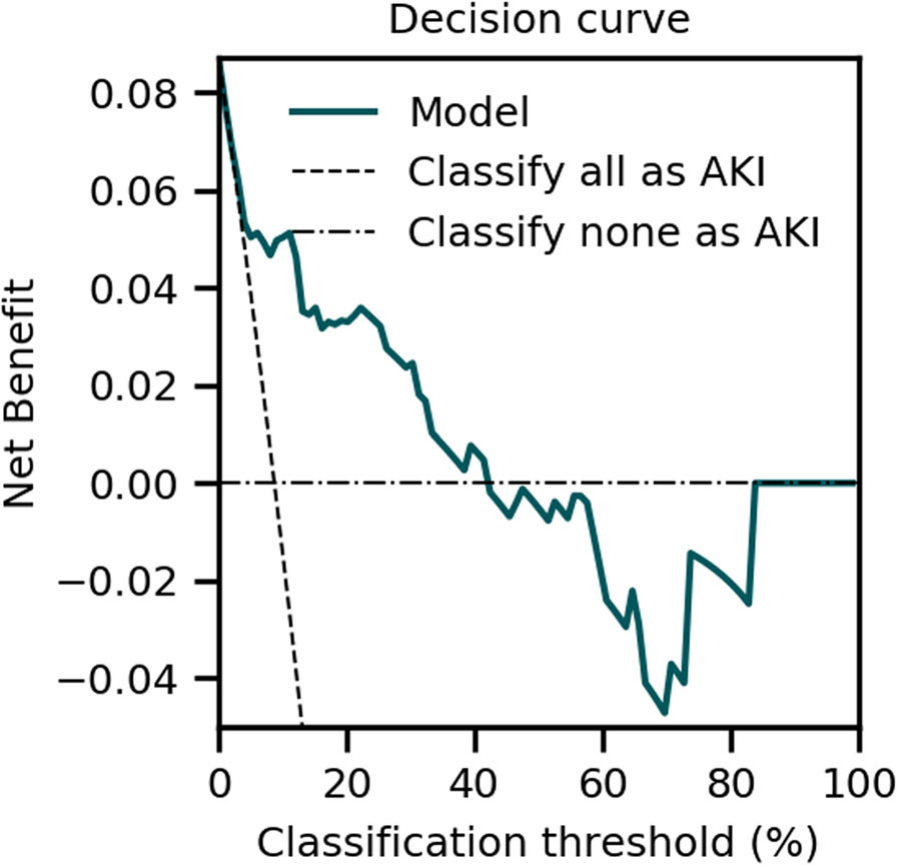
Illustration of the decision curve analysis. The example illustrates the decision curve of a model to predict whether patients will have AKI, from a population with an AKI prevalence of 9%. In the decision curve analysis, the classification threshold corresponds to the cutoff above which a patient is classified as “will develop AKI.” Knowing whether the patient will or will not have AKI will trigger different therapeutic interventions. Low classification thresholds are used when the associated therapy is not harmful; hence, patients will not suffer from being classified as false positives. High classification thresholds are used when the associated therapy is toxic or has side effects, and therefore, it is important to not classify patients as at risk for AKI when they are not (thereby limiting the number of false positive classifications). Currently, preventive measures for AKI are optimization of hemodynamics and prevention of nephrotoxicity, amenable to all patients and therefore corresponding to low classification thresholds. The net benefit is a weighted measure between true and false positives depending on the classification threshold [35]. The maximum net benefit is obtained by detecting all patients who will later develop AKI; therefore, this net benefit is the prevalence of AKI in the population (9%). The line corresponding to the trivial assumption that all patients will have AKI can be drawn (*classify all as AKI*, traditionally called *treat-all*). Similarly, the minimum net benefit is obtained by considering that no patient will develop AKI and is 0 (*classify none as AKI*, traditionally called *treat-none*). To be clinically useful, a model should have a higher net benefit than the two trivial classifications. Here, being slightly above the *classify all as AKI* curve, the model shows usefulness in the range 0–43%. Above 43%, the model shows negative net benefit, which reflects harm and should be avoided in clinical practice. Here, the model is clinically relevant as it shows benefit for low risk thresholds corresponding to its associated preventive measures

Discrimination, calibration, and clinical usefulness [30] were used to evaluate the performance of AKIpredictor and physicians. One hundred bootstrap samples were used to estimate confidence intervals on the performance metrics. Discrimination refers to how well the predictions allow to distinguish patients with and without AKI. Discrimination was evaluated with the receiver operating characteristic (ROC) curve and the area under the ROC curve (AUROC) [31]. Sensitivity, specificity, positive predictive value, and negative predictive value were reported using clinicians’ binary classifications. The DeLong test [32] from the pROC R package [33] was used for AUROC comparison. Calibration refers to the agreement between predicted probabilities and the observed frequency of AKI in the population. Calibration was assessed using calibration belts or curves where appropriate, together with the distribution of patient numbers [34]. Finally, the net benefit of the model was assessed by the difference between the expected benefit and the expected harm associated with AKI classification, as illustrated in Fig. 1. Clinical usefulness was visualized using decision curves and reported using ranges above *treat-all* and *treat-none* curves [35, 36]. An example of a decision curve with its interpretation is given in Fig. 1.

To assess the added value of the AKIpredictor to the predictions by physicians, a multivariable logistic regression was used to combine the estimated AKI risk by the AKIpredictor with the one by physicians.

## Results

### Study population

**Fig. 2:**
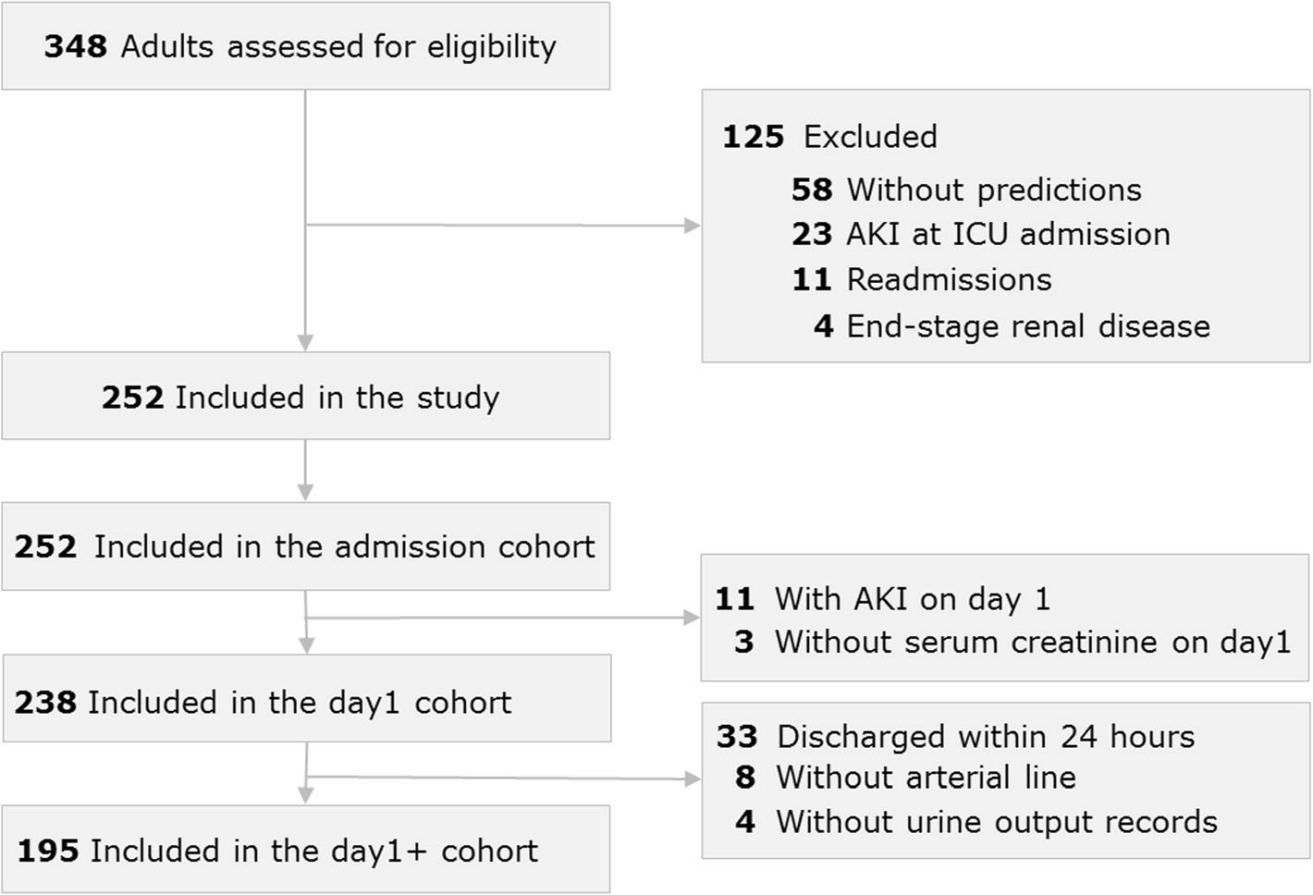
Flow chart

A total of 348 adults were considered for study inclusion, of which 58 were excluded because all prediction moments occurred during on-call time, 27 due to AKI or ESRD upon admission, and 11 due to readmission (Fig. 2). Two hundred and fifty-two patients remained for analysis.

Patients’ characteristics are reported in Table 2, for all patients, and Additional file 1: Table S1, for patients with predictions by physicians. Within the first week, 30 (12%) patients developed AKI-23 using both SCr and UO criteria and 23 (9%) with SCr criteria only. Baseline SCr was available in 202 (80.2%) patients and was calculated in the remaining 50 (19.8%).

**Table 2:** Patient characteristics and clinical outcomes

	Admission cohort	Day1 cohort	Day1+ cohort
*N*	252	238	195
AKI-23 by SCr and UO, *n* (%)	30 (12)	17 (7)	17 (9)
Time to AKI-23 by SCr and UO, hours from admission	27.1 (9.6–61.6)	39.7 (31.6–109.4)	39.7 (31.6–109.4)
AKI-23 by SCr, *n* (%)	23 (9)	13 (5)	13 (7)
Time to AKI-23 by SCr, hours from admission	28.3 (12.1–38.4)	37.1 (32.9–61.5)	37.1 (32.9–61.5)
ICU LOS, days	3 (2–7)	2 (2–6)	3 (2–7)
Demographics			
Age, year	65.5 (52.8–74.0)	65 (52–74)	66 (54–74)
Male gender, *n* (%)	155 (61.5)	146 (61.3)	121 (62.1)
Height, cm	171 (165–178)	171 (165–178)	171 (165–178)
Weight, kg	75.2 (65.0–86.3)	75.0 (65.0–86.0)	75.0 (63.5–85.7)
Diabetic, *n* (%)	6 (2.4)	5 (2.1)	3 (1.5)
Baseline SCr, mg/dL	0.88 (0.73–1.05)	0.88 (0.73–1.04)	0.88 (0.73–1.06)
Clinical parameters			
Elective admission, *n* (%)	154 (61.1)	149 (62.6)	124 (63.6)
Surgical category, *n* (%)			
Cardiac	103 (40.9)	98 (41.1)	85 (43.6)
Transplant	7 (2.8)	7 (2.9)	7 (3.6)
Others	92 (36.5)	86 (35.8)	71 (36.4)
Medical category, *n* (%)	50 (19.8)	47 (19.8)	32 (16.4)
Hemodynamic support at ICU admission, *n* (%)			
Pharmacological	165 (65.5)	158 (66.4)	140 (71.8)
Mechanical	4 (1.6)	3 (1.3)	3 (1.5)
Blood glucose at ICU admission, mg/dL	135 (113–155)	135 (114–155)	137 (116–160)
Sepsis upon ICU admission, *n* (%)	20 (7.9)	18 (7.6)	16 (8.2)
Maximum lactate on day 1, mg/dL	1.6 (1.1–2.4)	1.5 (1.1–2.3)	1.6 (1.1–2.4)
Bilirubin on day 1, mg/dL	0.54 (0.37–0.84)	0.54 (0.37–0.83)	0.53 (0.38–0.83)
Apache II score on day 1	14 (10–17)	13.5 (10–17)	14 (11–17)
SOFA score on day 1	9 (5–11)	9 (5–11)	9 (6–12)
SCr on day 1, md/dL,	0.87 (0.68–1.08)	0.87 (0.68–1.05)	0.88 (0.70–1.08)
Monitoring parameters^a^			
UO slope, mL/hour	−0.00014 (−0.00052 to 0.00034)	−0.00013 (−0.00051 to 0.00035)	−0.00014 (−0.00046 to 0.00032)
Total amount of UO, mL/hour	1025 (770–1437)	1047 (789–1451)	1048 (495–1473)
Blood pressure below 60 mmHg, min	10 (3–50)	10 (3–48)	11 (4–51)
Blood pressure above average, min	644 (569–696)	647 (569–698)	655 (593–716)
Dose of vasopressors, mg	2.7 (0–8.9)	2.7 (0–8.6)	4.3 (0–9.7)

Data are reported as median (IQR) unless otherwise indicated

*Abbreviations*: *AKI* acute kidney injury, *ICU* intensive care unit, *IQR* interquartile ranges, *LOS* length of stay, *SCr* serum creatinine, *UO* urine output

^a^Measured during the first 24 h of ICU stay

### AKIpredictor predictions

Predictions were calculated in 252 patients at admission, in 238 patients on the first morning, and in 195 patients after 24 h (Fig. 2).

When classifying AKI by SCr, the AKIpredictor predicted AKI-23 with AUROC [95% CI] 0.78 [0.69–0.88], 0.94 [0.91–0.98], 0.93 [0.88–0.97], and net benefit in ranges 0–74%, 0–48%, and 3–43% respectively upon admission, on day 1, and after 24 h (Additional file 1: Figure S1).

**Fig. 3:**
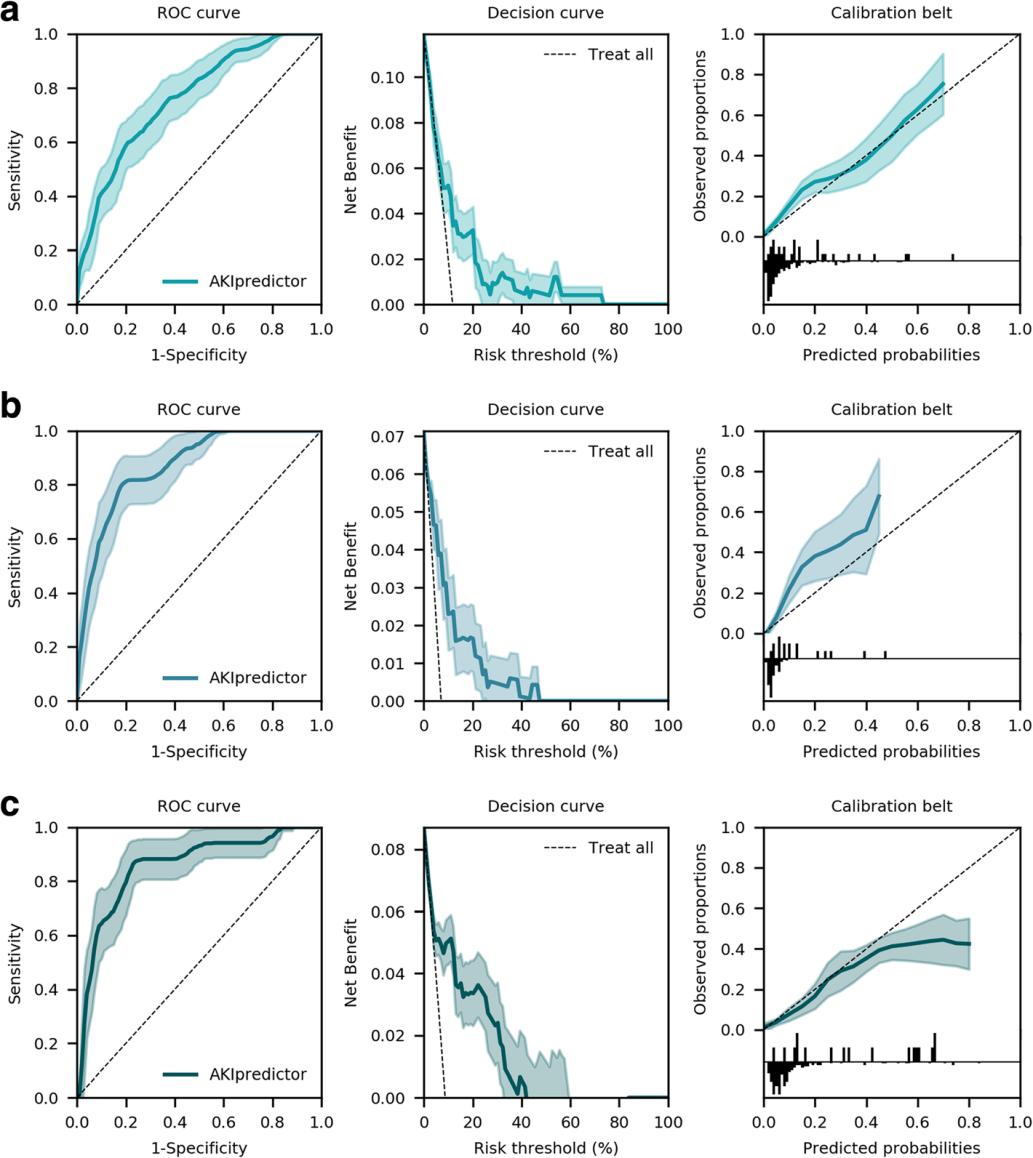
Performance of AKIpredictor for predictions of AKI-23 by SCr and UO. **a** At ICU admission (*n* = 252), AUROC [95% CI] was 0.76 [0.66–0.85], net benefit in ranges 0–74%. **b** On the first morning of ICU stay (*n* = 238), AUROC [95% CI] was 0.87 [0.79–0.95], net benefit in ranges 0–48%. **c** After 24 h (*n* = 195), AUROC [95% CI] was 0.85 [0.75–0.96], net benefit in ranges 0–43%

When classifying AKI by SCr and UO criteria, the AKIpredictor predicted AKI-23 with AUROC [95% CI] 0.76 [0.66–0.85], 0.87 [0.79–0.95], and 0.85 [0.75–0.96] and net benefit in ranges 0–74%, 0–48%, and 0–43% respectively upon admission, on day 1, and after 24 h (Fig. 3).

### Physicians’ predictions

Questionnaires were filled by 43 physicians, of whom 24 (55.8%) juniors and 19 (44.2%) seniors (Additional file 1: Table S2). Seven hundred nine predictions were collected (Additional file 1: Table S3): 183 predictions about 120 patients upon admission, 394 predictions about 187 patients on the morning of the first day, and 128 predictions about 89 patients after 24 h. Although the protocol allowed gathering predictions 1 h before the time point, the majority was obtained later (Additional file 1: Table S3, 183 (100%) on admission, 383 (97.2%) on the first morning, 77 (60.2%) at 24 h). On average, predictions were obtained 68 min after ICU admission, 140 min after the first morning, and 20 min after 24 h. Additional file 1: Table S4 presents the physicians’ predictions by confidence level and seniority level. Overall, confidence obtained at later time points was higher, with 53 (29%) highly confident predictions at admission, 147 (37.3%) on the first morning, and 55 (43%) after 24 h.

**Fig. 4:**
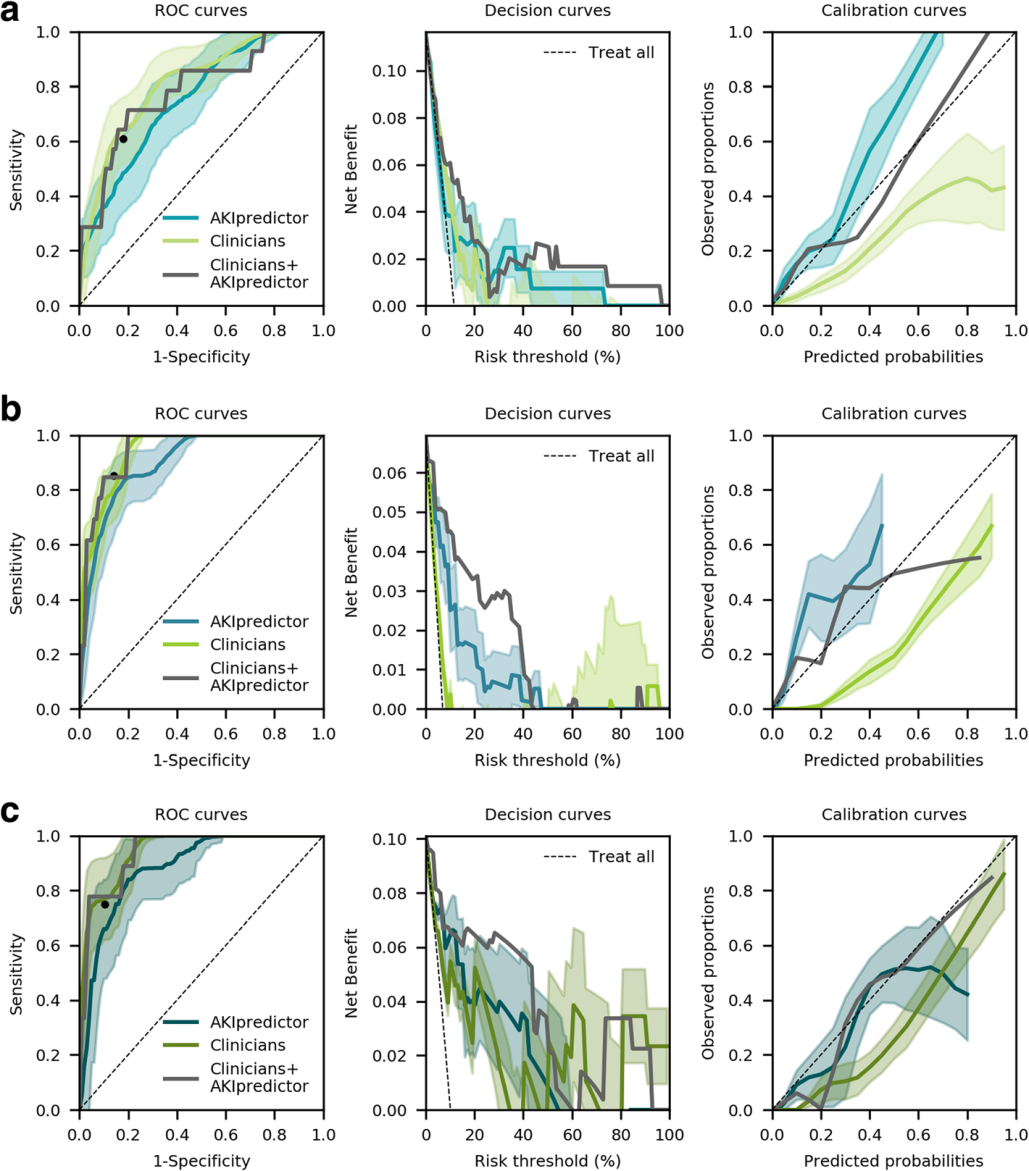
Comparison of performance of AKIpredictor and physicians for prediction of AKI-23 by SCr and UO. The black dot represents the classification threshold from the physicians. **a** At ICU admission (*n* = 120), AUROCs [95% CI] were 0.80 [0.69–0.92] and 0.75 [0.62–0.88] (*P* = 0.25), net benefit in ranges 0–26% and 0–74% for clinicians, and AKIpredictor respectively. Physicians’ classification threshold achieved 55% sensitivity, 82% specificity, 33% positive predictive value, and 94% negative predictive value. **b** On the first morning of ICU stay (*n* = 187), AUROCs [95% CI] were 0.94 [0.89–0.98] and 0.89 [0.82–0.97] (*P* = 0.27), net benefit in ranges (0–10% + 90–96%) and (0–48%) for clinicians and AKIpredictor respectively. Physicians’ classification threshold achieved 85% sensitivity, 86% specificity, 31% positive predictive value, and 99% negative predictive value. **c** After 24 h (*n* = 89), AUROCs [95% CI] were 0.95 [0.89–1.00] and 0.89 [0.79–0.99] (*P* = 0.09), with net benefit in ranges (0–36% + 40–48% + 50–67% + 80–100%) and (0–58%) for clinicians and AKIpredictor respectively. Clinicians’ classification threshold achieved 75% sensitivity, 90% specificity, 43% positive predictive value, and 97% negative predictive value. The wide confidence interval for high risk thresholds on the decision curve is amenable to the low number of patients. Therefore, findings should be interpreted with caution

Physicians predicted AKI with AUROC [95% CI] 0.80 [0.69–0.92], 0.94 [0.89–0.98], and 0.95 [0.89–1.00] and net benefit in ranges (0–26%), (0–10% + 90–96%), and (0–36% + 40–48% + 50–67% + 80–100%) respectively upon admission, on the first morning, and after 24 h (Fig. 4). Additional file 1: Figure S2 shows performance when using physicians’ binary predictions, which allowed for the identification of the classification threshold they adopted: sensitivity and specificity were respectively 55% and 82% on admission, 85% and 86% on day 1, and 75% and 90% after 24 h. As compared to juniors, senior physicians showed higher discrimination and calibration at all time points (Additional file 1: Figure S3, AUROC 0.81 vs 0.85 at admission; AUROC 0.87 vs 0.92 on day 1; AUROC 0.90 vs 0.96 at 24 h for juniors and seniors respectively). Finally, when physicians expressed low or medium confidence in their predictions, their performance was worse (Additional file 1: Figure S4, AUROC 0.74 versus 0.85 at admission, AUROC 0.93 vs 0.92 on day 1, and AUROC 0.89 versus 0.98 at 24 h, for low-medium versus high confidence respectively).

In the subset of patients with physicians’ predictions (Fig. 4), AKIpredictor predicted AKI with AUROC [95% CI] 0.75 [0.62–0.88] (*P* = 0.25 as compared to clinicians) with net benefit in ranges 0–74%, higher than physicians in ranges 14–74%. On day 1, AUROC [95% CI] was 0.89 [0.82–0.97] (*P* = 0.27) with higher net benefit compared with physicians in ranges 0–48%. Finally, after 24 h, AUROC [95% CI] was 0.89 [0.79–0.99] (*P* = 0.09) with higher net benefit compared with physicians in ranges 0–20% + 23–50%. The wide confidence interval for high risk thresholds on the decision curve (Fig. 4c) is amenable to the low number of patients. Therefore, findings should be interpreted with caution.

### Combining AKIpredictor with physicians’ predictions

In the subset of patients where physicians’ predictions were combined with the AKIpredictor, no improvement in discriminability was observed as compared to physicians (*P* = 0.96, 0.39, and 0.41 respectively for admission, day 1, and after 24 h), but a better calibration resulted in wider and higher ranges of net benefit at all time points (Additional file 1: Figure S5). The same was observed for junior physicians only (Additional file 1: Figure S6) and for low-medium confidence predictions (Additional file 1: Figure S7).

## Discussion

In this study, we compared the performance of the AKI risk estimated by physicians versus the one provided by AKIpredictor, a machine-learning-based clinical prediction model [19]. The comparison was made at three different time points: upon ICU admission, on the first morning in ICU, and after 24 h of ICU stay. There was no statistically significant difference in discrimination between physicians and AKIpredictor at any time point. However, on average, physicians provided predictions later than the AKIpredictor.

Decision curve analysis helps to identify the expected benefit or harm when performing classification at different risk levels. Compared to physicians, the AKIpredictor showed improved net benefit for AKI classification thresholds above 26% upon admission and for almost all ranges of AKI classification thresholds on day 1 and after 24 h. This comparison provides meaningful insight on how the tool could be used in clinical practice.

As shown by the calibration curve, physicians tend to overestimate the risk of AKI. In the decision curve, this behavior results in a net benefit similar to considering that all patients will develop AKI (*treat-all* curve) [37]. Currently, there is no treatment for AKI and preventive measures are mainly supportive, so there would be no harm from misclassifying a patient as high risk (false positive). However, if only high-risk patients were needed for a clinical trial or if a new, potentially toxic or expensive therapy for AKI became available, only the AKIpredictor would be able to identify the correct patients, limiting selection bias, unnecessary exposure, or higher costs. This situation corresponds to a high classification threshold for AKI, for which only the AKIpredictor showed net benefit.

Additional clinical implications of the AKIpredictor were highlighted by this study. First, it allows a consistent stratification of patients, with similar performance to a well-trained clinical staff. Second, it provides predictions at fixed time points without delays, while physicians required on average more time. Third, although senior physicians are best at predicting AKI, they have to supervise a higher number of patients and might benefit from an electronic warning system that draws their attention to patients at risk. Fourth, when doctors are in doubt and express a low or medium confidence in their predictions, these predictions are actually less performant. In such cases, they might find it useful to consult the AKIpredictor.

This study is the first prospective validation study of the AKIpredictor. Compared to the results of the original development study (AUROC [95% CI] 0.77 [0.77–0.77], 0.80[0.80–0.80], and 0.82 [0.82–0.82] for admission, day1, and day1+ predictions) [19], the models showed similar performance upon admission and an even higher performance on the first morning and after 24 h. Wider confidence intervals have been observed due to the limited sample size. This observed improvement might be explained by the difference in patient population (sicker patients with more comorbidities in the development population). Indeed, in the original study, the AKIpredictor had a slightly worse performance in septic patients. By not predicting during on-call time, a lower proportion of patients with an unplanned admission, such as sepsis, were included. The difference in population might also explain the lower prevalence of severe AKI (9% vs 12%) in this cohort. Furthermore, the design of the current study might have raised clinicians’ awareness towards the kidney, which in turn could have prevented AKI development and hence affected AKI incidence.

It is striking that, although the AKI predictor was developed to predict AKI based on SCr and not UO criteria, in this study, the model performed well, even while AKI was defined by both SCr and UO.

### Strengths and limitations

This study has several strengths. First, it is prospective in design and hence detailed in data collection. Second, in order to reduce bias from lack of collaboration by physicians, interviewers made efforts to obtain questionnaires for all included patients: predictions for all but 12 patients were gathered for at least one time point. In addition, when feasible, predictions were obtained from both junior and senior physicians, allowing for secondary analysis based on physicians’ experience. Finally, to the best of our knowledge, this study is the first of its kind to assess physicians’ estimation of AKI risk, which provides benchmarking opportunities for comparison against other AKI prognosticators such as biomarkers.

This study had the following limitations: first, as a single-center study with limited sample size, findings have to be used with caution, as they might not generalize to other centers. In particular, fewer predictions were available on admission and after 24 h. Second, a bias in favor of clinicians cannot be excluded as (1) the AKIpredictor is not optimized to predict AKI defined by UO as in the development study the definition of AKI was only based on the SCr criterion [19]. Additionally, due to the low prevalence of AKI-23 after 24 h of ICU stay, no model was developed [19]. Therefore, at 24 h, the comparison is made using the AKIpredictor model for AKI-123. (2) Physicians received 3 more hours to provide their predictions. Therefore, they had access to later clinical information than the AKIpredictor. (3) Physicians did not provide predictions at all time points. However, we limited this bias by asking predictions for at least one time point in all but 12 patients. Third, predictions from junior and senior physicians were not available for all patients. Therefore, when averaging predictions based on physicians’ experience and level of confidence, performance did not improve although the separate analysis clearly showed a difference in performance on both levels. Fourth, the presence of interviewers only during office hours favored the collection of predictions about elective patients over emergent ones, and this may explain the low rate of AKI observed. A sensible power calculation was unfortunately not available to begin with, due to the lack of studies investigating physicians’ predictive abilities, but ours could represent a benchmark for future ones. Finally, the questionnaire did not include the reason behind clinicians’ predictions. This precludes any comparison between how physicians and the AKIpredictor made the predictions.

## Conclusion

Physicians can predict AKI with good discrimination, but tend to overestimate the risk of AKI, pointing to a poor calibration in the low-risk patients. The AKIpredictor performed on par with physicians in terms of discrimination and did better in terms of calibration and net benefit. This highlights the potential uses of the AKIpredictor in clinical practice: selection of high-risk patients or reducing false positives in studies evaluating new and potentially harmful therapies. Additionally, our findings suggest a potential for overall improvement of care with the concurrent use of physicians’ expertise and the AKIpredictor. Due to the limited sample size, external validation and further prospective studies of the AKIpredictor are warranted, in particular to compare how physicians and the algorithm made predictions.

## Additional file

Additional file 1: Supplementary methods: Physician's predictions. **Figure S1.** Performance of AKIpredictor for prediction of AKI-23 by serum creatinine. **Figure S2.** Performance of binary predictions by physicians. **Figure S3.** Performance of clinicians split by seniority level. **Figure S4.** Performance of clinicians split by confidence level. **Figure S5.** Comparison of performance of AKIpredictor, physicians and their combination. **Figure S6.** Comparison of performance of junior physicians and the combination of junior physicians with AKIpredictor. **Figure S7.** Comparison of performance of physicians with low-medium confidence in their predictions and the combination of their predictions with AKIpredictor. **Table S1.** Patient characteristics and clinical outcomes for patients with predictions by physicians and AKIpredictor. **Table S2.** Physicians’ generalities. **Table S3.** Description of physicians’ predictions. **Table S4.** Description of physicians’ predictions per seniority and confidence levels. **Appendix A.** prediction questionnaire. **Appendix B.** Physician questionnaire Tables S1, S2, S3, and S4 and Figures S1, S2, S3, S4, S5, S6, and S7. (DOCX 2070 kb)

## Data Availability

The datasets generated and analyzed during the current study are not publicly available due to no prior agreement with the local ethical committee. Open reasonable request, amendment can be requested to the corresponding author to share the necessary data.

## Abbreviations

AKI: Acute kidney injury
AKI-123: Any stage of AKI
AKI-23: The most severe stages of AKI
AUROC: Area under the receiver operating characteristic
ESRD: End-stage renal disease
FWO: Research Foundation Flanders
ICU: Intensive care unit
IQR: Interquartile range
KDIGO: Kidney Disease Improving Global Outcomes
LOS: Length of stay
ROC: Receiver operating characteristic
SCr: Serum creatinine
SD: Standard deviation
UO: Urine output
