# Supplementary material for: Machine learning versus physicians’ prediction of acute kidney injury in critically ill adults: a prospective evaluation of the AKIpredictor
Source: Crit Care. 2019 Aug 16;23:282. doi: 10.1186/s13054-019-2563-x (PMC6697946; doi:10.1186/s13054-019-2563-x)
Supplement: Supplementary file 1 — Supplementary methods: Physician's predictions. Figure S1. Performance of AKIpredictor for prediction of AKI-23 by serum creatinine. Figure S2. Performance of binary predictions by physicians. Figure S3. Performance of clinicians split by seniority level. Figure S4. Performance of clinicians split by confidence level. Figure S5. Comparison of performance of AKIpredictor, physicians and their combination. Figure S6. Comparison of performance of junior physicians and the combination of junior physicians with AKIpredictor. Figure S7. Comparison of performance of physicians with low-medium confidence in their predictions and the combination of their predictions with AKIpredictor. Table S1. Patient characteristics and clinical outcomes for patients with predictions by physicians and AKIpredictor. Table S2. Physicians’ generalities. Table S3. Description of physicians’ predictions. Table S4. Description of physicians’ predictions per seniority and confidence levels. Appendix A. prediction questionnaire. Appendix B. Physician questionnaire Tables S1, S2, S3, and S4 and Figures S1, S2, S3, S4, S5, S6, and S7. (DOCX 2070 kb) [file 13054_2019_2563_MOESM1_ESM.docx]

Machine learning versus physicians’ prediction of acute kidney injury in critically ill adults
A prospective evaluation of the AKIpredictor

Additional file

Marine Flechet, PhD*; Stefano Falini^[[1]](#footnote-1)^, MD*; Claudia Bonetti^[[2]](#footnote-2)^; Fabian Güiza, PhD; Miet Schetz, PhD; Greet Van den Berghe, PhD; Geert Meyfroidt, PhD

* authors contributed equally

Clinical Division and Laboratory of Intensive Care Medicine, Academic Department of Cellular and Molecular Medicine, KU Leuven, Leuven, Herestraat 49, B-3000 Leuven, Belgium

**Corresponding author:**

Geert Meyfroidt, MD, PhD

E-mail: geert.meyfroidt@uzleuven.be

University of Leuven

Department of Intensive Care Medicine

Herestraat 49; B-3000 Leuven; Belgium

Phone +32 16 344021

Fax +32 16 344015

**Conflict of interest**: On behalf of all authors, the corresponding author states that there is no conflict of interest.

# Supplementary methods

## Physicians’ predictions

Junior residents are in training for their basic specialty (anesthesiology, internal medicine, or pediatrics), and have at least 3 years of experience as a physician. Senior residents are physicians in training for intensive care medicine, and have already obtained their basic specialty. All staff members are certified specialists in intensive care medicine.

# Supplementary results

## Supplementary figures

**
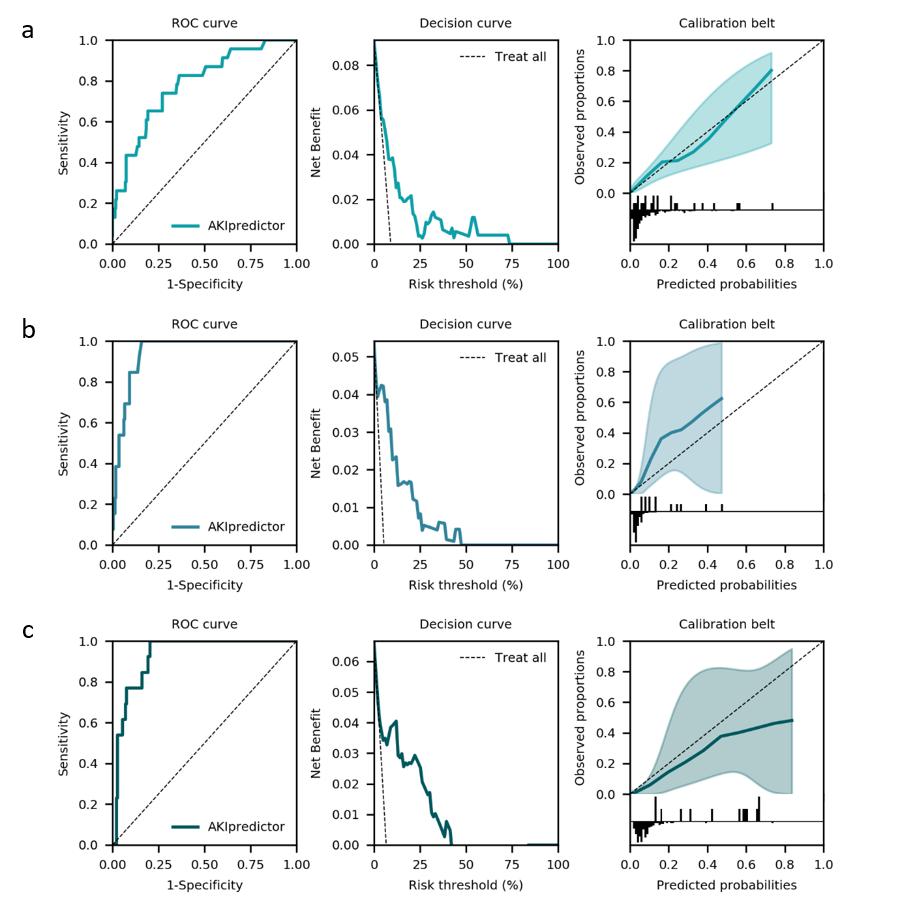
**

**Figure S1.** Performance of AKIpredictor for prediction of AKI-23 by serum creatinine
(a) At ICU admission (n=252), AUROC [95% CI] 0.78 [0.69-0.88], net benefit in ranges (0-74%). Optimal cutoff was 8%; (b) On the first morning of ICU stay (n=240), AUROC [95% CI] 0.94 [0.91-0.98], net benefit in ranges (0-48%). Optimal cutoff was 6%; (c) After 24 hours (n=195), AUROC [95% CI] 0.93 [0.88-0.97], net benefit in ranges (3-43%). Optimal cutoff was 12%.


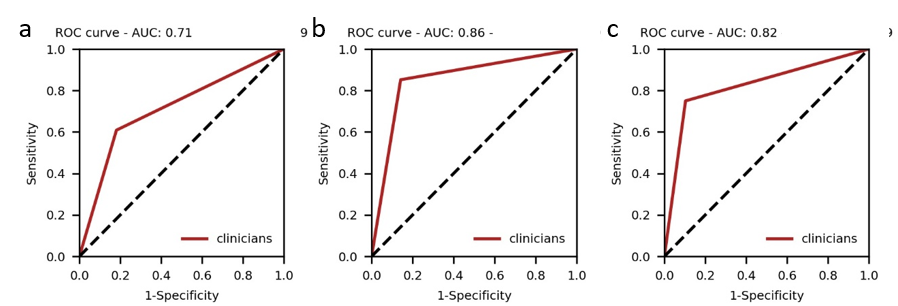


**Figure S2**. Performance of binary predictions by physicians.
(a) Upon ICU admission, AUROC [95%CI], 0.71 [0.60-0.82] (n=183). Physicians’ classification threshold achieved 55% sensitivity, 82% specificity, 33% positive predictive value and 94% negative predictive value. (b) On day 1, AUROC [95% CI], 0.86 [0.78-0.93] (n=394). Physicians’ classification threshold achieved 85% sensitivity, 86% specificity, 31% positive predictive value and 99% negative predictive value. (c) After 24 hours, AUROC [95% CI], 0.82 [0.69-0.95] (n=128). Physicians’ classification threshold achieved 75% sensitivity, 90% specificity, 43% positive predictive value and 97% negative predictive value.


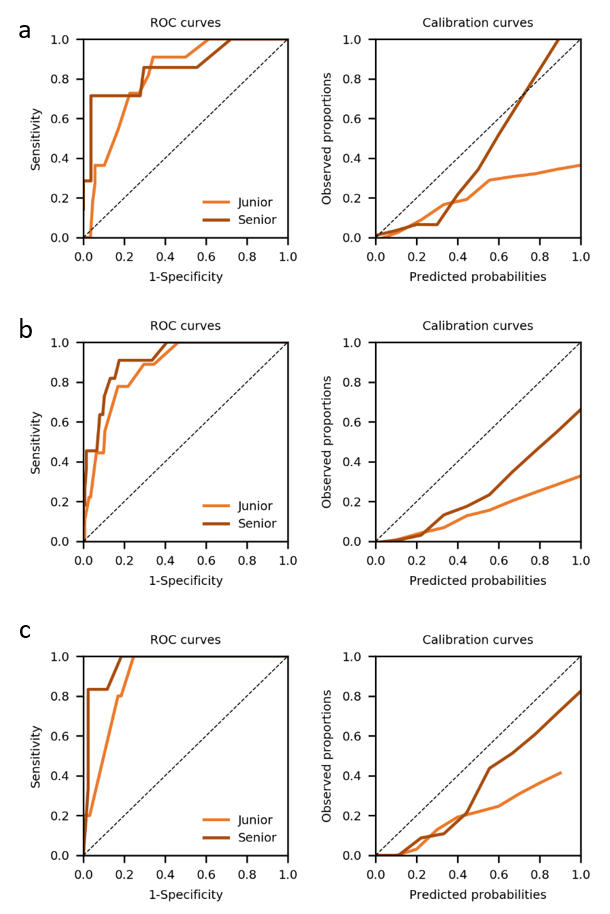


**Figure S3**. Performance of clinicians split by seniority level.
(a) Upon admission, AUROCs were 0.81 and 0.85, for junior and senior respectively. Classification threshold had 55% sensitivity and 75% specificity for junior, 71% sensitivity and 94% specificity for senior. (b) On day 1, AUROCs were 0.87 and 0.92, for junior and senior respectively. Classification threshold had 78% sensitivity and 82% specificity for junior, 91% sensitivity and 84% specificity for senior. (c) After 24 hours, AUROC were 0.90 and 0.96, for junior and senior respectively. Classification threshold had 60% sensitivity and 88% specificity for junior, 83% sensitivity and 91% specificity for senior. Decision curves are not represented, as they are not comparable (different patient population).


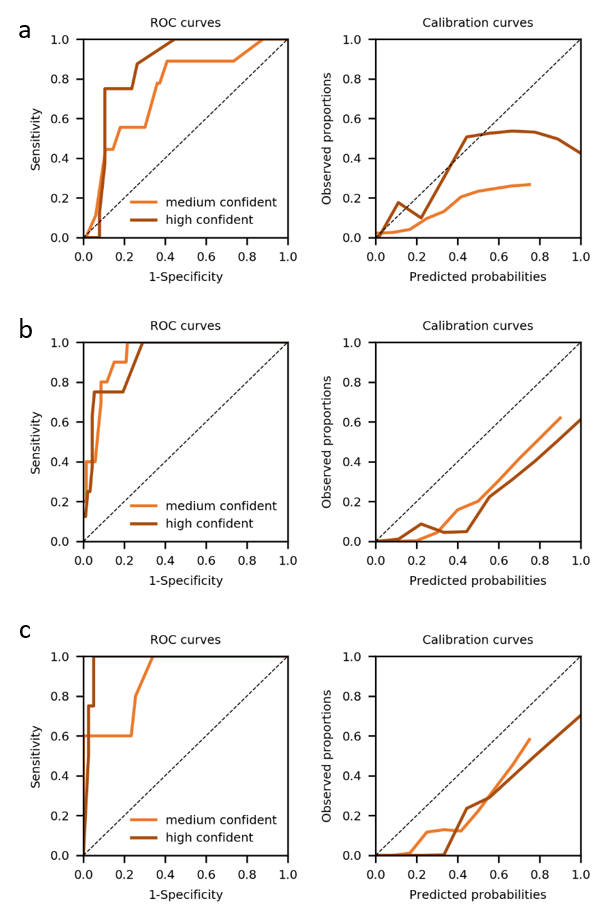


**Figure S4**. Performance of clinicians split by confidence level.
Low confident predictions had too few occurrences and were combined to medium confident predictions. (a) Upon admission, AUROCs were 0.74 and 0.85, for medium and high confidence respectively. Classification threshold had 78% sensitivity and 64% specificity for medium confidence, 75% sensitivity and 89% specificity for high confidence. (b) On day 1, AUROCs were 0.93 and 0.92, for medium and high confidence respectively. Classification threshold had 90% sensitivity and 85% specificity for medium confidence, 75% sensitivity and 95% specificity for high confidence. (c) After 24 hours, AUROC were 0.89 and 0.98, for medium and high confidence respectively. Classification threshold had 80% sensitivity and 74% specificity for medium confidence, 60% sensitivity and 95% specificity for high confidence. Decision curves are not represented, as they are not comparable (different patient population).


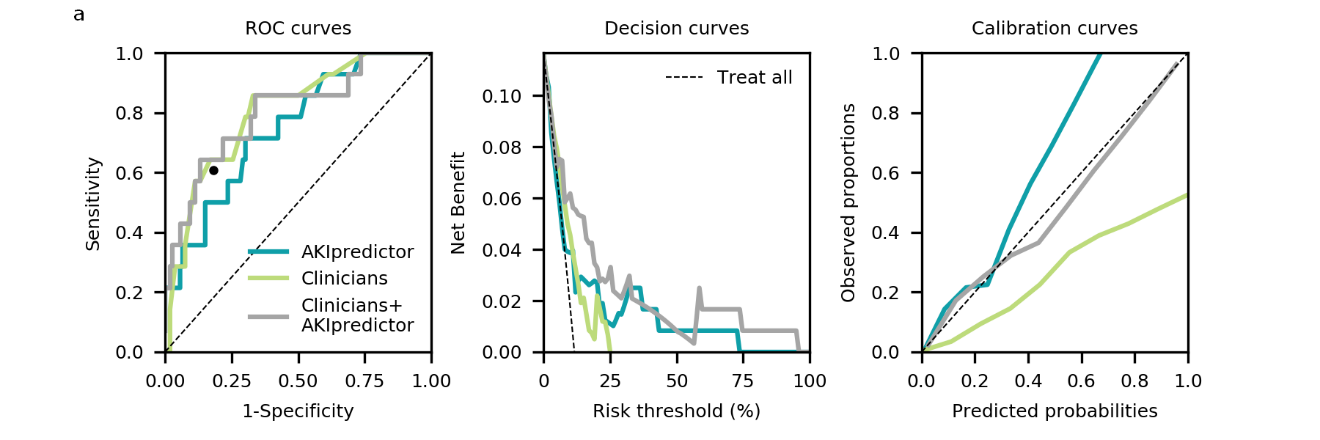

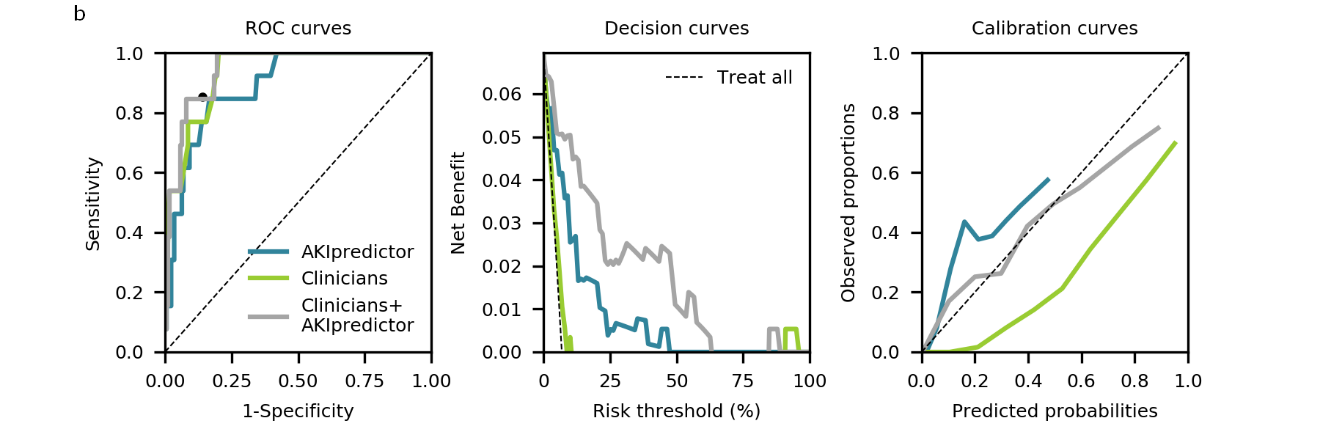

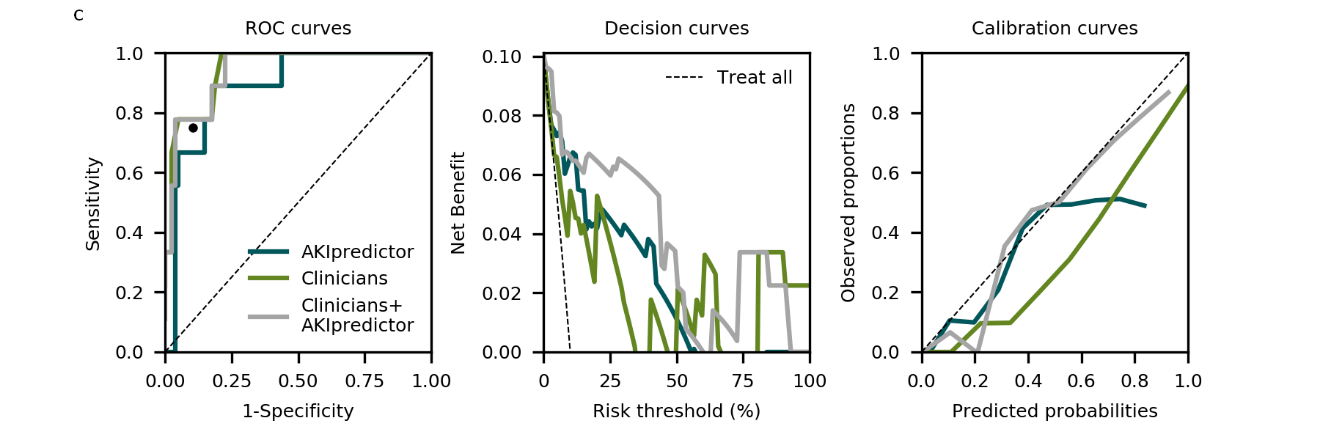


**Figure S5**. Comparison of performance of AKIpredictor, physicians and their combination.
The black dot represents the classification threshold from the clinicians. (a) At ICU admission (n=120), AUROCs [95% CI] were 0.80 [0.69-0.92], 0.75 [0.62-0.88] (P=0.25 as compared to physicians), 0.80 [0.67-0.94] (P=0.96 as compared to physicians), net benefit in ranges (0-26%), (0-74%), (0-96%) for clinicians, AKIpredictor, and their combination respectively. (b) On the first morning of ICU stay (n=187), AUROCs [95% CI] were 0.94 [0.89-0.98], 0.89 [0.82-0.97] (P=0.27 as compared to physicians), 0.95 [0.90-0.99] (P=0.39 as compared to physicians), net benefit in ranges (0-10+90-96%), (0-48%), (0-64%+84-89%) for clinicians, AKIpredictor, and their combination respectively. (c) After 24 hours (n=89), AUROCs [95% CI] were 0.95 [0.89-1.00], 0.89 [0.79-0.99] (P=0.09 as compared to physicians), 0.94 [0.88-1.00] (P=0.41 as compared to physicians), with net benefit in ranges (0-36%+40-48%+50-67%+80-100%), (0-58%), (0-61%+63-93%) for clinicians, AKIpredictor, and their combination respectively.


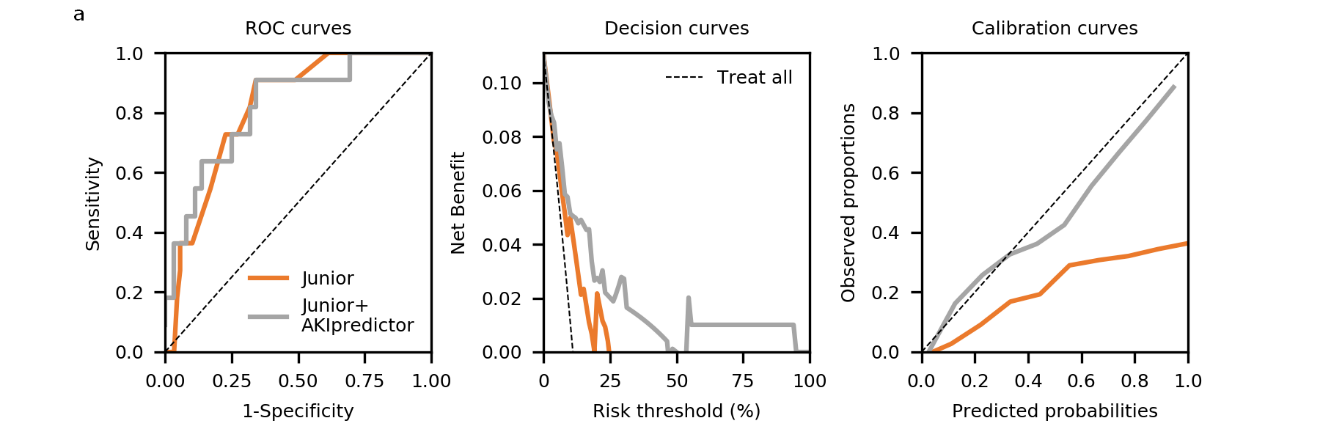


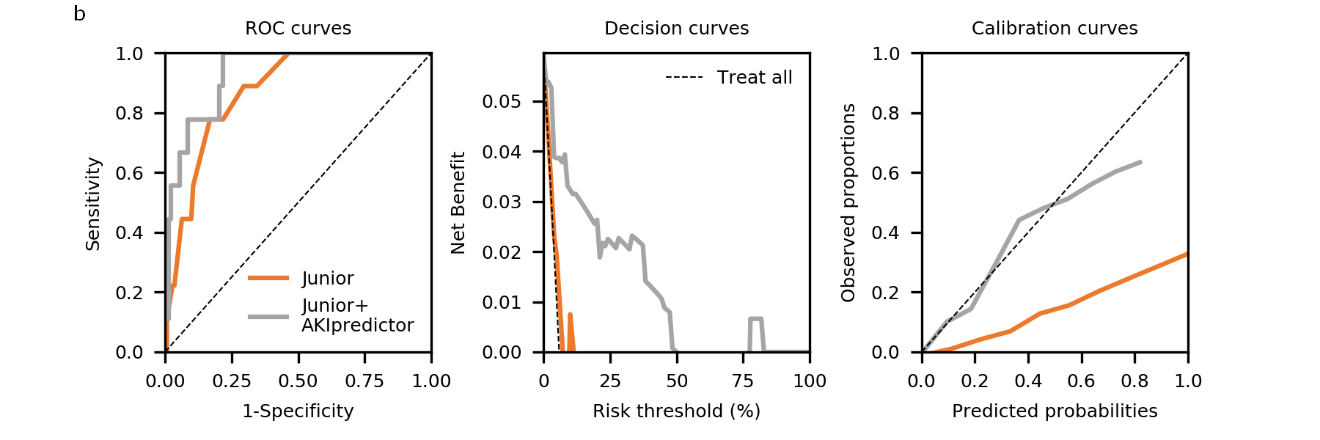


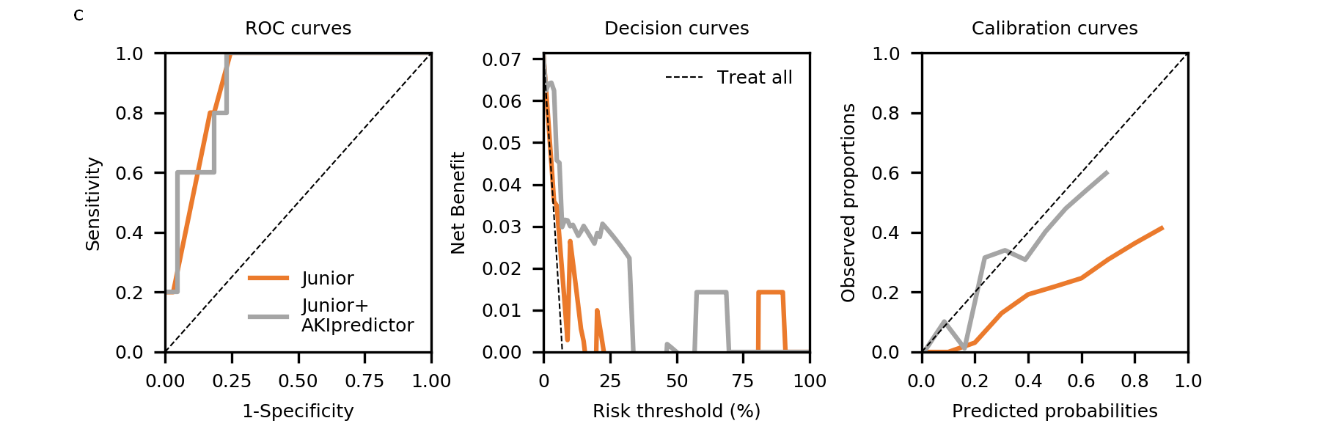


**Figure S6**. Comparison of performance of junior physicians and the combination of junior physicians with AKIpredictor.
A logistic regression is used to combine the predictions of junior physicians and of the AKIpredictor. Consequently, an optimal calibration is obtained and the associated net benefit represents the maximum net benefit that could be achieved by the junior physicians using the AKIpredictor.
(a) At ICU admission (n=99), AUROCs were 81.8, 81.8, net benefit in ranges (0-26%), (4-48%+54-95%) for junior physicians and their combination with AKIpredictor respectively. (b) On the first morning of ICU stay (n=151), AUROCs were 91.4, 93.0, net benefit in ranges (10-13%), (0-51%+77-83%) for junior physicians, and their combination with AKIpredictor respectively. (c) After 24 hours (n=70), AUROCs were 90.5, 89.8, with net benefit in ranges (0-17%+20-24%+80-91%), (0-35%+46-51%+57-70%), for junior physicians, and their combination with AKIpredictor respectively


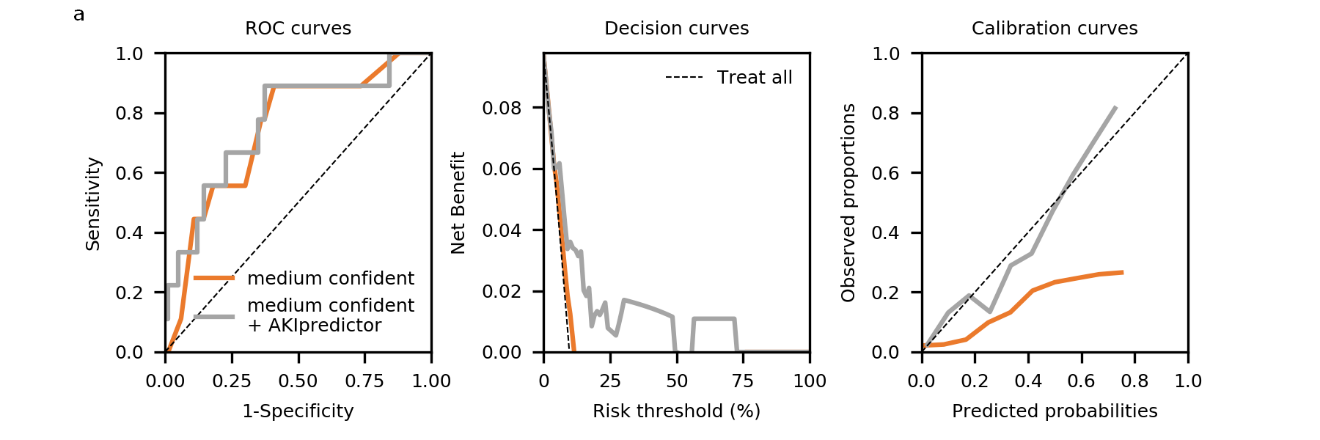


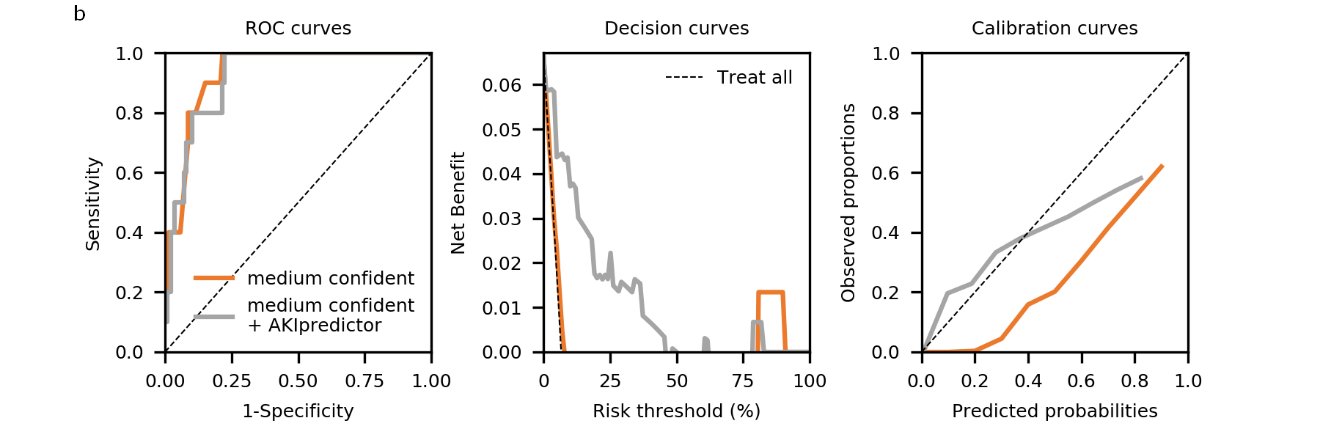


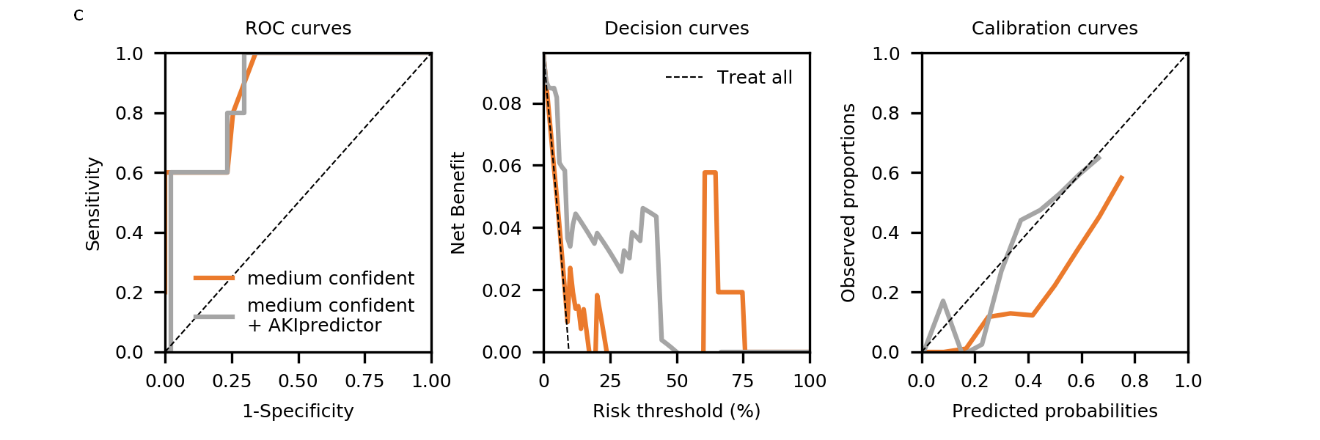


**Figure S7**. Comparison of performance of physicians with low-medium confidence in their predictions and the combination of their predictions with AKIpredictor.
A logistic regression is used to combine the low-medium confidence predictions with the ones from the AKIpredictor. Consequently, an optimal calibration is obtained and the associated net benefit represents the maximum net benefit that could be achieved using the AKIpredictor.
(a) At ICU admission (n=99), AUROCs were 74.9, 76.4, net benefit in ranges (0-13%), (4-51%+56-73%) for low-medium confidence predictions and their combination with AKIpredictor respectively. (b) On the first morning of ICU stay (n=151), AUROCs were 90.9, 92.2, net benefit in ranges (0-9%+80-91%), (0-51%+60-63%+78-83%) for low-medium confidence predictions, and their combination with AKIpredictor respectively. (c) After 24 hours (n=70), AUROCs were 88.3, 88.1, with net benefit in ranges (0-19%+20-25%+60-76%), (0-51%), for junior physicians, and their combination with AKIpredictor respectively.

## Supplementary tables

**Table S1**. Patient characteristics and clinical outcomes for patients with predictions by physicians and AKIpredictor.

|  | Admission cohort | Day1 cohort | Day1+ cohort |
| --- | --- | --- | --- |
| N | 120 | 187 | 89 |
| AKI-23 by SCr and UO, n (%) | 14 (12) | 13 (7) | 9 (10) |
| Time to AKI-23 by SCr and UO, hours from admission | 37.3 (16.0-98.0) | 58.6 (31.6-109.4) | 58.6 (32.9-61.7) |
| AKI-23 by SCr, n (%) | 11 (9) | 10 (5) | 8 (9) |
| Time to AKI-23 by SCr, hours from admission | 16.3 (13.0-37.3) | 49.1 (31.9-61.6) | 49.1 (32.6-61.5) |
| ICU LOS, days | 2.5 (1-6) | 2(1.5-6) | 3 (2-7) |
| **Demographics** |  |  |  |
| Age, year | 66 (51-74) | 64 (52-74) | 67 (53-74) |
| Male gender, n (%) | 72 (60.0) | 123 (65.8) | 55 (61.8) |
| Height, cm | 172 (165-178) | 172 (165-179) | 171 (163-178) |
| Weight, kg | 75 (67-88) | 76 (65-86) | 74 (65-86) |
| Diabetic, n (%) | 2 (1.7) | 4 (2.1) | 0 (0) |
| Baseline SCr, mg/dL | 0.87 (0.73-1.05) | 0.88 (0.74-1.05) | 0.92 (0.71-1.08) |
| **Clinical parameters** |  |  |  |
| Elective admission, n (%) | 75 (62.5) | 122 (65.2) | 60 (67.4) |
| Surgical category, n (%) |  |  |  |
| Cardiac | 53 (44.2) | 81 (43.3) | 43 (48.3) |
| Transplant | 6 (5.0) | 4 (2.1) | 4 (4.5) |
| Others | 45 (38.3) | 61 (32.3) | 28 (31.5) |
| Medical category, n (%) | 15 (12.5) | 41 (21.9) | 14 (15.7) |
| Hemodynamic support at ICU admission, n (%) |  |  |  |
| Pharmacological | 81 (67.5) | 131 (70.1) | 66 (74.2) |
| Mechanical | 1 (0.8) | 3 (1.6) | 2 (2.2) |
| Blood glucose at ICU admission, mg/dL | 134 (115-158) | 135 (116-153) | 138 (113-159) |
| Sepsis upon ICU admission, n (%) | 6 (5.0) | 11 (5.8) | 2 (2.2) |
| Maximum lactate on day 1, mg/dL | 1.4 (1.0-2.2) | 1.5 (1.1-2.3) | 1.6 (1.1-2.4) |
| Bilirubin on day 1, mg/dL | 0.55 (0.35-0.86) | 0.54 (0.38-0.80) | 0.49 (0.36-0.79) |
| Apache II score on day 1 | 14 (10-17) | 13 (10-17) | 15 (11-17) |
| SOFA score on day 1 | 9 (4-11) | 9 (5-11) | 10 (7-12) |
| SCr on day1, md/dL | 0.88 (0.71-1.10) | 0.87 (0.71-1.03) | 0.89 (0.72-1.14) |
| **Monitoring parameters^a^** |  |  |  |
| UO slope, ml/hour | -0.00014 (-0.0006 to 0.00046) | -0.00015 (-0.0005 to 0.00034) | -0.00021 (-0.00062 to 0.00031) |
| Total amount of UO, ml/hour | 1130 (903-1537) | 1035 (770-1458) | 1077 (788-1515) |
| Blood pressure below 60 mmHg, min | 9 (2-38) | 10 (3-51) | 12 (4-86) |
| Blood pressure above average, min | 647 (569-707) | 647 (561-695) | 658 (585-708) |
| Dose of vasopressors, mg | 2.7 (0-8.9) | 3.5 (0-8.9) | 4.3 (0-9.8) |

^a^  measured during first 24 hours of ICU stay.
Data are reported as median (IQR) unless otherwise indicated. Abbreviations: AKI, acute kidney injury; ICU, intensive care unit; IQR, interquartile ranges; LOS, length of stay; SCr, serum creatinine; UO, urine output.

**Table S2**. Physicians’ generalities

|  | Physicians |
| --- | --- |
| N | 43 |
| Age, years, median (IQR) | 30 (29-34) |
| Male gender, n (%) | 25 (58.1) |
| Seniority level, n (%) |  |
| Junior resident | 24 (55.8) |
| Senior resident | 8 (18.6) |
| Staff member | 11 (25.6) |
| ICU experience, year, median (IQR) | 1 (0.5-2.5) |

**Table S3.** Description of physicians’ predictions

|  | Admission | Day1 | Day1+ |
| --- | --- | --- | --- |
| N patients | 120 | 187 | 89 |
| N predictions | 183 | 394 | 128 |
| Self-filled predictions, n (%) | 18 (9.8) | 4 (1.0) | 16 (12.5) |
| Predictions collected by interviewer, n (%) | 165 (90.2) | 390 (99.0) | 112 (87.5) |
| Predictions collected before time point, n (%) | 0 (0) | 11 (2.8) | 51 (39.8) |
| Predictions collected after time point, n (%) | 183 (100) | 383 (97.2) | 77 (60.2) |
| Time between prediction and time point, min, median (IQR) | 68 (36-105) | 140 (120-150) | 20 (-26 to 83) |

**Table S4**. Description of physicians’ predictions per seniority and confidence levels

|  | Admission | | | Day1 | | | Day1+ | | |
| --- | --- | --- | --- | --- | --- | --- | --- | --- | --- |
|  | Junior | Senior | Staff | Junior | Senior | Staff | Junior | Senior | Staff |
| N total | 104 | 50 | 29 | 157 | 106 | 131 | 70 | 34 | 24 |
| N (%)  low confident | 4 (3.8) | 0 (0.0) | 6 (20.7) | 8 (5.0) | 2 (1.9) | 4 (3.0) | 3 (4.3) | 0 (0.0) | 0 (0.0) |
| N (%) medium confident | 65 (62.5) | 40 (80.0) | 15 (51.7) | 94 (59.9) | 71 (67.0) | 68 (51.9) | 35 (50.0) | 21 (61.8) | 14 (58.3) |
| N (%)  very confident | 35 (33.7) | 10 (20.0) | 8 (27.6) | 55 (35.0) | 33 (31.1) | 59 (45.0) | 32 (45.7) | 13 (38.2) | 10 (41.7) |

# Appendix A: Prediction questionnaire


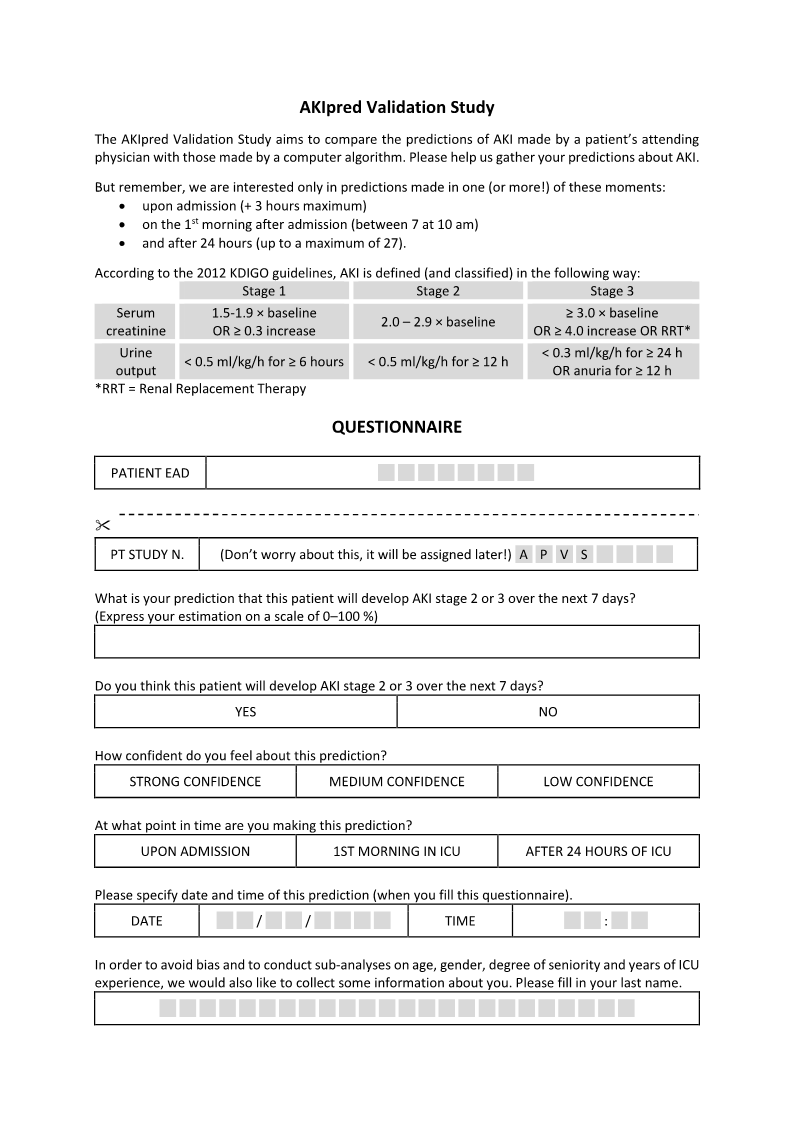


# Appendix B : Physician questionnaire


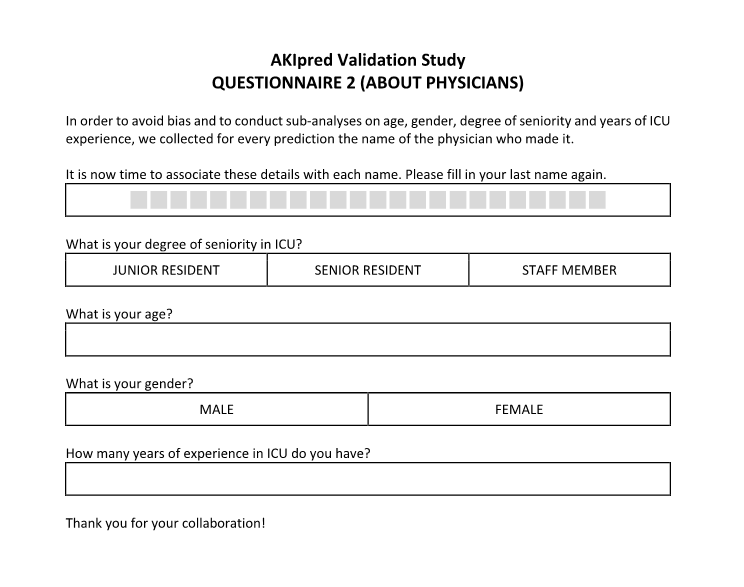


1. S. Falini is now at Department of Anesthesia and General Intensive Care, Humanitas Clinical and Research Center, via Manzoni 56, 20089 Rozzano (MI), Italy [↑](#footnote-ref-1)
2. C. Bonetti is now at University of Milano-Bicocca, Piazza dell'Ateneo Nuovo 1, 20126 Milan, Italy [↑](#footnote-ref-2)
